# Clinical Characteristics and Outcomes of Patients with Ischemic and Non-Ischemic Complete Heart Block

**DOI:** 10.7759/cureus.1244

**Published:** 2017-05-12

**Authors:** Murtaza Sundhu, Mehmet Yildiz, Mubbasher Syed, Bhavan Shah, Sajjad Gul, Omer Afzal, Lon Castle

**Affiliations:** 1 Internal Medicine Residency, Fairview Hospital, Cleveland Clinic, USA; 2 Electrophysiology, Fairview Hospital, Cleveland Clinic, USA

**Keywords:** heart block, complete heart block, cardiology, permanent pacemaker, myocardial infarction, third degree, electrophysiology, syncope

## Abstract

**Introduction:**

Third degree or complete heart block (CHB) is a medical emergency and usually requires permanent pacemaker placement. CHB can be caused by cardiac ischemia or non-ischemic conditions such as infiltrative diseases or fibrosis. The purpose of this study is to identify the baseline clinical characteristics associated with ischemic versus non-ischemic causes of complete heart block and compare their outcomes.

**Materials and methods:**

An institutional review broad approval was granted by the Cleveland Clinic Health System. In our study, 216 patients with CHB presenting to Fairview Hospital between January 2014 and June 2016 were initially identified using the International Classification of Disease (ICD) codes at discharge. Only the patients with a new diagnosis of complete heart block (CHB) were included in the study (total N=62), which led to the exclusion of 154 patients. The patients were characterized into non-ischemic and ischemic groups based on cardiac marker elevation, electrocardiogram changes and/or cardiac catheterization findings. In all the patients, data including the following was collected: demographics such as age, gender, ethnicity and body mass index; pre-existing comorbidities such as hypertension, chronic kidney disease, diabetes mellitus, thyroid disease, previous coronary artery disease, history of cancer; use of nodal blocking agents, electrolyte abnormalities on admission, echocardiographic parameters such as ejection fraction (EF), right ventricular systolic pressure (RVSP), left ventricular end diastolic and systolic volumes (LVEDV and LVESV, respectively). The primary outcome was all-cause mortality and the secondary outcome was pacemaker placement. Categorical variables were analyzed using chi-square and continuous variables using ANOVA.

**Results:**

Out of 62 patients (N=62), 48 had non-ischemic and 14 had ischemic CHB. The mean age was 67 years (95% CI, 60.55-74.73) in the ischemic group and 75 years (95% CI, 71.52-78.80) in the non-ischemic group, p=0.04. Patients with ischemic CHB had a lower mean EF of 49.6% (95% CI, 42.04%-57.23%) compared to 57.42% in non-ischemic CHB patients (95% CI, 53.97%-60.87%), p=0.05. History of coronary artery disease was present in 71.4% (10/14) patients with ischemic CHB compared to 37.5% (18/48) patients with non-ischemic CHB, p=0.02. There was no statistically significant difference between the two groups in terms of gender, diabetes, hypertension, thyroid dysfunction, chronic kidney disease, nodal blocking agents, electrolyte abnormalities or smoking status. For outcomes, 6/48 (12.5%) of patients with non-ischemic CHB had died compared to 3/14 (21.4%) ischemic CHB (p=0.327). Permanent pacemaker was implanted in 45/48 patients (93.75%) of the non-ischemic CHB compared to 6/14 (42.83%) in the ischemic group (p<0.001).

**Conclusions:**

Patients with ischemic CHB are younger, and they have a lower ejection fraction but they are less likely to get a pacemaker compared to non-ischemic CHB. Further studies with a bigger sample size are required to understand the long term mortality outcomes of patients with CHB.

## Introduction

Third degree heart block is the complete block or dissociation between the atria and the ventricles and a condition when none of the atrial impulses reach the ventricle. The causes of the third degree heart block in children could be congenital [1] or familial [2] and in adults is related to ischemia (of atrioventricular node) or could be non-ischemic including increased vagal tone, fibrosis (Lenegre's disease in patients with age <60) [3], sclerosis (Lev's disease in patients with age >70) of the conduction system [4], electrolyte abnormalities, infiltrative diseases or iatrogenic including nodal blocking medication, cardiac surgery, catheter ablations, and transcatheter aortic valve implantation.

The clinical presentation depends on the level of the block and the escape rhythm that develops as a result [5]. The escape rhythm depends on the automaticity or intrinsic rate of the tissue distil to the block [6]. The escape rhythms can be divided into narrow complex or wide complex escape rhythm based on the QRS duration (whether the QRS duration is less than or more than 120 milliseconds). Junctional or atrioventricular (AV) nodal escape rhythm develops, which is a narrow complex, when the block is within the atrioventricular node or at the level of His bundle. The escape rhythm is a wide QRS complex when the block is below the His bundle (infraHisian) [7-8].

They may be asymptomatic in very few number of cases. The presenting complains mostly include weakness, dizziness, palpitation, dyspnea, or syncope [5]. Diagnosis of the third degree heart block can be made by a 12-lead electrocardiogram and usually requires permanent pacemaker as treatment.

In this study, we try to understand the clinical characteristics pertaining to new onset CHB and carefully analyze the outcomes in ischemic and non-ischemic CHB patients as there is no comparison between these groups.

## Materials and methods

### Study design

This is a retrospective observational cohort of patients admitted to the Fairview hospital from January 2014 to June 2016 with third degree heart block. The diagnosis was confirmed by an electrocardiogram by a cardiologist. The study was approved by the institutional review board of the Cleveland Clinic. Individual consent was waived because of the retrospective nature of the study.

The patients were initially identified using the International Classification of Disease (ICD) codes applied at discharge including both ICD-9 and ICD-10 codes as there was a transition during the requested time frame. In our study, 216 patients who had a third degree heart block in their billing codes were initially identified. Patients with the age >18 years and a new diagnosis of the third degree heart block were included in the study. Patients with a pre-existing/known history of CHB and congenital heart disease were excluded from the study. This led to the exclusion of 154 patients. Data was collected by reviewing the electronic medical records. The ischemic and non-ischemic CHB were differentiated by cardiac markers elevation, ischemic electrocardiogram changes and/or cardiac catheterization. The differentiation was made by the cardiologists.

### Data collection

We collected data for the following: demographics including age, gender, ethnicity and body mass index; pre-existing comorbidities including diabetes mellitus, hypertension, previous coronary artery disease, history of cardiac surgery, acute kidney injury on admission, chronic kidney disease, thyroid disease, history of cancer; use of nodal blocking agents, electrolyte abnormalities on admission and echocardiographic findings including ejection fraction, right ventricular systolic pressure (RVSP), left ventricular end diastolic and systolic volumes. The primary outcome was all-cause mortality while the secondary outcome was permanent pacemaker placement. Outcomes were assessed by follow-up in the office or last pacemaker check. During follow-up, other parameters like percentage of right ventricular pacing, underlying rhythm, battery status of the pacemaker, sensing parameters, and follow-up echocardiogram findings including ejection fraction and RVSP were also collected.

### Statistical analysis

Data analysis was performed using IBM SPSS Statistics Version 23 (IBM Corp, NY, USA). The categorical variables were compared using Fisher’s exact method as there were cells with less than count of five. The continuous variables were compared using analysis of variance (ANOVA). An analysis of covariance (ANCOVA) model was created to assess the difference in initial ejection fraction and the follow-up ejection fraction to see if there was any statistically significanct difference between both groups.

## Results

Among the 62 patients (N=62) that met the inclusion criteria, 48 had non-ischemic complete heart block and 14 had ischemic compete heart block. Out of all the patients, 69% (43/62) were males, 37% (23/62) had diabetes mellitus, 6.4% (4/62) had hypothyroidism, 79% (49/62) had hypertension, 25.8% (16/62) had chronic kidney disease, 45.2% (28/62) had a history of coronary artery disease, 25.8% (16/62) had history of cancer, 51.6% (32/62) were on nodal blocking agents, 11.3% (7/62) were current smokers, 56.6% (35/62) were former smokers, and 32.6% (20/62) had never smoked (Table 1).

**Table 1:** Baseline Categorical Variables

Baseline Categorical Variables				
Variables	Total (N=62)	Ischemic (N=14)	Non-ischemic (N=48)	P - Value
Male	43 (69.4%)	12 (85.7%)	31 (64.6%)	0.12
Female	19 (30.6%)	2 (14.3%)	17 (35.4%)	
Diabetes Present	23 (37.1%)	5 (35.7%)	18 (37.5%)	0.58
Hypertension Present	49 (79.1%)	11 (78.6%)	38 (79.2%)	0.61
History of thyroid Disease	4 (6.4%)	0 (0%)	4 (8.3%)	0.34
History of Coronary Artery Disease Present	28 (45.2%)	10 (71.4%)	18 (37.5%)	0.02
History of Chronic Kidney Disease Present	16 (25.8%)	4 (28.6%)	12 (25.0%)	0.52
History of Cancer Present	16 (25.8%)	3 (21.4%)	13 (27.1%)	0.48
Current Smoker	7 (11.2%)	1 (7.1%)	6 (12.5%)	0.76
Former Smoker	35 (56.5%)	9 (64.3%)	26 (54.2%)	
Never smoked	20 (32.3%)	4 (28.6%)	16 (33.3%)	
Nodal Blocking agent prescribed	32 (51.6%)	9 (64.3%)	23 (47.9%)	0.22
Nodal Blocking agents Not prescribed	30 (48.4%)	5 (35.7%)	25 (52.1%)	

The mean age in the group with ischemic complete heart block was 67.64 years (95% CI, 60.56-74.73) and 75.60 years (95% CI, 72.00-79.21) in non-ischemic CHB (p=0.04). The mean ejection fraction in ischemic CHB group was 49.6% (95% CI, 42.05%-57.23%) compared to 57.25% (95% CI, 53.74%-60.76%) in non-ischemic CHB patients (p=0.05). A history of coronary artery disease was present in 71.4% (10/14) patients with ischemic CHB compared to 37.5% (18/48) patients with non-ischemic CHB (p=0.02). The right ventricular systolic pressure (RVSP) was 33.13 (95% CI, 21.71 - 44.54) in ischemic CHB versus 41.87 (95% CI, 36.50-47.25) in non-ischemic CHB (p=0.17), which was not statistically significant (Table 2).

**Table 2:** Continuous Variables

Continuous Variables								
		N	Mean	Std. Deviation	Std. Error	95% Confidence Interval for Mean		P – Value
						Lower Bound	Upper Bound	
Age	Ischemic	14	67.64	12.28	3.28	60.56	74.73	0.04
	Non-Ischemic	48	75.60	12.42	1.79	72.00	79.21	
	Total	62	73.81	12.73	1.62	70.57	77.04	
Body Mass Index	Ischemic	12	29.50	6.72	1.94	25.23	33.77	0.36
	Non-Ischemic	46	27.67	5.98	0.88	25.89	29.45	
	Total	58	28.05	6.13	0.80	26.44	29.66	
Potassium	Ischemic	13	4.09	0.45	0.13	3.81	4.36	0.10
	Non-Ischemic	48	4.37	0.59	0.08	4.20	4.54	
	Total	61	4.31	0.57	0.07	4.16	4.46	
Magnesium	Ischemic	13	2.07	0.27	0.07	1.91	2.23	0.76
	Non-Ischemic	46	2.05	0.26	0.04	1.97	2.13	
	Total	59	2.05	0.26	0.03	1.99	2.12	
Ejection Fraction (EF)	Ischemic	11	49.64	11.30	3.41	42.05	57.23	0.05
	Non-Ischemic	44	57.25	11.55	1.74	53.74	60.76	
	Total	55	55.73	11.80	1.59	52.54	58.92	
Right Ventricular Systolic Pressure (RVSP)	Ischemic	8	33.13	13.65	4.83	21.71	44.54	0.17
	Non-Ischemic	39	41.87	16.58	2.66	36.50	47.25	
	Total	47	40.38	16.32	2.38	35.59	45.18	
EF at follow up	Ischemic	3	46.67	18.93	10.93	-0.36	93.69	0.80
	Non-Ischemic	17	44.41	13.84	3.36	37.30	51.53	
	Total	20	44.75	14.13	3.16	38.14	51.36	
RVSP at follow up	Ischemic	3	39.67	23.25	13.42	-18.08	97.41	0.66
	Non-Ischemic	13	35.15	14.23	3.95	26.56	43.75	
	Total	16	36.00	15.41	3.85	27.79	44.21	

**Figure 1:**
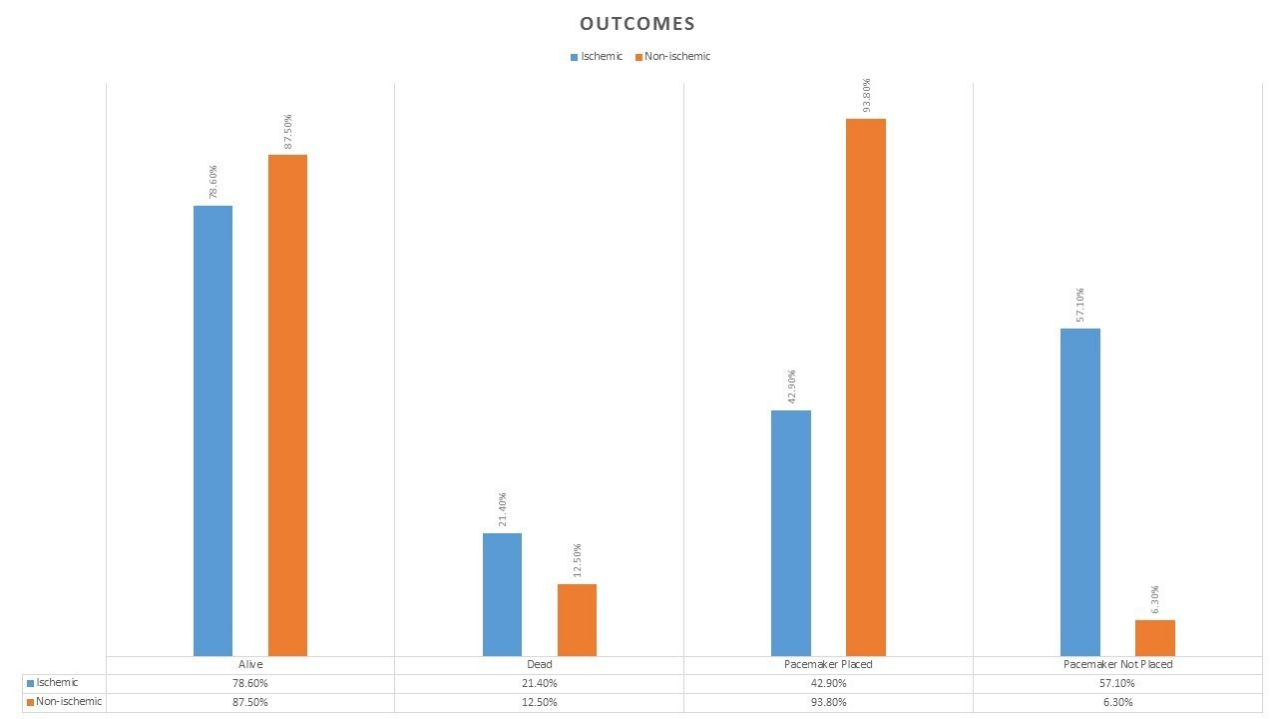
Outcomes Graph

**Figure 2:**
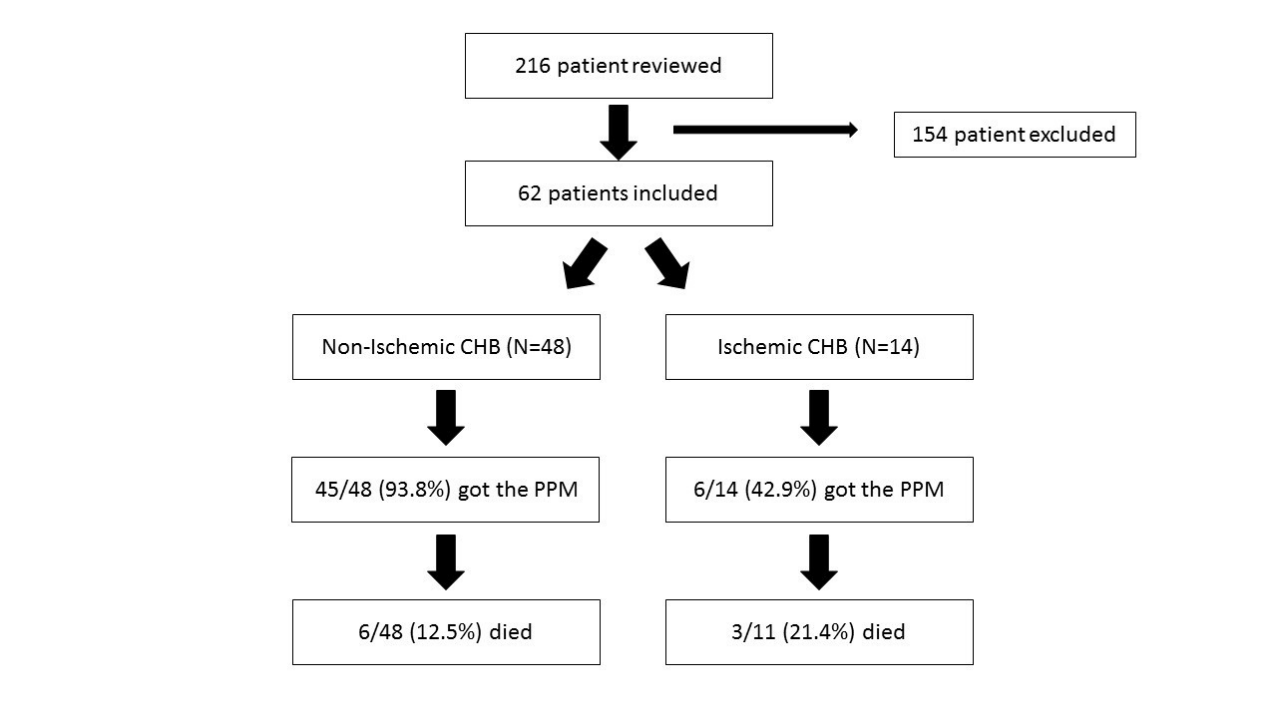
Overview

For outcomes, 45/48 (93.75%) patients with non-ischemic CHB had the permanent pacemaker implanted versus 6/14 (42.83%) patients with ischemic CHB (p<0.01). The primary outcome of death was reported in 6/48 (12.5%) patients with non-ischemic CHB compared to 3/14 (21.4%) patients in ischemic CHB group (p=0.327) (Table 3 and Figure 1). The overview of the study is provided in Figure 2.

**Table 3:** Outcomes

Variable	Total (N=62)	Ischemic (N=14)	Non-ischemic (N=48)	P Value
Primary Outcome				
Alive	53 (85.5%)	11 (78.6%)	42 (87.5%)	0.327
Dead	9 (15.5%)	3 (21.4%)	6 (12.5%)	
Secondary Outcome				
Pacemaker Placed	51 (82.3%)	6 (42.9%)	45 (93.8%)	<0.01
Pacemaker Not Placed	11 (17.7%)	8 (57.1%)	3 (6.3%)	

Further, an ANCOVA model was created to assess the difference between both groups in terms of the initial and follow-up ejection fraction (EF). Follow-up EF was reported for 20 patients out of whom three had ischemic CHB and 17 had non-ischemic CHB. There was no statistical difference (p=0.909) in the means of the EF difference at the time of diagnosis and follow-up visit.

## Discussion

Adult-acquired (non-ischemic) complete heart block has a poor outcome without a permanent pacemaker [9]. Patients who are symptomatic do especially worse, and patients who get pacemakers do very well [9]. A permanent pacemaker is indicated when no reversible cause for complete heart block is identified [10]. Non-ischemic third degree heart block is hypothesized to be more frequent in patients with thyroid disease and diabetes [11]. In our study, there was no difference between the ischemic and non-ischemic complete heart block with regards to diabetes or thyroid disease as suggested previously [11].

The incidence of ischemic complete heart block after acute myocardial infarction has reduced [12-13]. The prognosis of acute myocardial infarction complicated by complete heart block (CHB) is poor and in-hospital death rates are significantly higher compared to patients with myocardial infarctions not associated with CHB [12, 14].

Ischemic and non-ischemic complete heart blocks are dealt as separate entities and we tried to study them head to head. We found no difference in terms of the pre-existing comorbidities including diabetes mellitus, hypothyroidism, hypertension, use of nodal blocking agents or chronic kidney disease. However, the patients with ischemic CHB are younger and frequently have a previous history of coronary artery disease compared to non-ischemic CHB patients. The ejection fraction is also lower in the ischemic CHB group, which is expected because of active myocardial ischemia/infarction. The right ventricular systolic pressure was higher in patients with non-ischemic CHB, which might suggest some relationship with pulmonary hypertension, but this difference was not statistically significant.

In terms of the outcomes, the patients with ischemic CHB are less likely to get a permanent pacemaker, and the difference was statistically significant. This is because most of the CHB after myocardial ischemia resolves spontaneously. The patients with ischemic CHB died more frequently compared to the patients in the non-ischemic group, but this difference was not statistically significant. This suggests that the patients with ischemic CHB do worse than the CHB patients without ischemia.

## Conclusions

Patients with ischemic complete heart block are less likely to get a pacemaker. They are younger with a lower ejection fraction at presentation and have a more frequent history of coronary artery disease. We need more studies with a bigger sample size to establish the mortality difference between them.
